# The Deubiquitinating Enzyme USP48 Interacts with the Retinal Degeneration-Associated Proteins UNC119a and ARL3

**DOI:** 10.3390/ijms232012527

**Published:** 2022-10-19

**Authors:** Laura Sánchez-Bellver, Andrea Férriz-Gordillo, Marc Carrillo-Pz, Laura Rabanal, Francesc R. Garcia-Gonzalo, Gemma Marfany

**Affiliations:** 1 Department of Genetics, Microbiology and Statistics, Universitat de Barcelona, Avda. Diagonal 643, 08028 Barcelona, Spain; 2 Centro de Investigación Biomédica en Red de Enfermedades Raras (CIBERER), Instituto de Salud Carlos III, 28029 Madrid, Spain; 3 Departamento de Bioquímica, Facultad de Medicina, Universidad Autónoma de Madrid, 28029 Madrid, Spain; 4 Instituto de Investigaciones Biomédicas “Alberto Sols”, Consejo Superior de Investigaciones Científicas (CSIC), 28029 Madrid, Spain; 5 Instituto de Investigación Hospital Universitario La Paz (IdiPAZ), 28029 Madrid, Spain; 6 Institut de Biomedicina-Institut de Recerca Sant Joan de Déu (IBUB-IRSJD), Universitat de Barcelona, 08028 Barcelona, Spain; 7 DBGen Ocular Genomics, 08028 Barcelona, Spain

**Keywords:** USP48, retina, inherited retinal dystrophies, cilium, ciliopathies

## Abstract

Proteins related to the ubiquitin-proteasome system play an important role during the differentiation and ciliogenesis of photoreceptor cells. Mutations in several genes involved in ubiquitination and proteostasis have been identified as causative of inherited retinal dystrophies (IRDs) and ciliopathies. USP48 is a deubiquitinating enzyme whose role in the retina is still unexplored although previous studies indicate its relevance for neurosensory organs. In this work, we describe that a pool of endogenous USP48 localises to the basal body in retinal cells and provide data that supports the function of USP48 in the photoreceptor cilium. We also demonstrate that USP48 interacts with the IRD-associated proteins ARL3 and UNC119a, and stabilise their protein levels using different mechanisms. Our results suggest that USP48 may act in the regulation/stabilisation of key ciliary proteins for photoreceptor function, in the modulation of intracellular protein transport, and in ciliary trafficking to the photoreceptor outer segment.

## 1. Introduction

The vertebrate retina, the light-sensitive neurosensory tissue that lines the inner surface of the eye, is arranged in ordered layers of different neuronal types. Rods and cones, the two types of photoreceptor cells, are the neurons in charge of capturing photons and transforming the light stimuli into an electrical signal that is transmitted to the brain through the optic nerve [1]. Photoreceptors possess a highly polarised morphology where all proteins required for photoreception and phototransduction are localised at the most apical compartment of the cell, the outer segment (OS). The OS is a specialised neurosensory cilium whose structure is maximised for photon capture as it contains tightly packed membranous discs ordered in stacks and filled with opsins, the photopigments. However, the OS lacks all the protein synthesis and metabolism machinery, thereby, OS components are synthesised in the inner segment (IS) of photoreceptors and transported to the OS through the connecting cilium (CC), a microtubular extension that acts as a ciliary gate [2,3]. As the distal membranous disks are daily shed and, consequently, the OS is continuously being renewed, there is a high demand on protein synthesis for its regeneration, hence the need for correct and efficient delivery of OS components to the cilium.

Inherited retinal dystrophies (IRDs) are a broad group of clinically and genetically heterogeneous neurodegenerative disorders characterised by progressive—and sometimes complete—vision loss, a major cause of untreatable blindness worldwide [4,5]. Among other molecular causes, retinopathies can result from dysfunction of the cilium, known as retinal ciliopathies, accounting for one-fourth of IRDs [6,7]. As cilia are ubiquitous organelles involved in a broad range of cellular functions, retinal ciliopathies are often associated with other co-morbidities in syndromic retinal ciliopathies [3,8]. In turn, cilium defects can be originated from mutations in genes encoding an extensive variety of proteins involved in ciliary structure, function, signalling, biogenesis, or transport [9,10,11,12,13], and this explains the high genetic and clinical heterogeneity, frequent lack of genotype-phenotype correlations, and difficulties in the molecular diagnosis of ciliopathies.

Cilium formation and maintenance are extremely regulated processes that highly depend on ubiquitin-proteasome system-related proteins, such as E3 ligases and deubiquitinating (DUB) enzymes [14,15]. In fact, post-translational ubiquitin and ubiquitin-like modifications play an important role during photoreceptor differentiation and ciliogenesis. Indeed, mutations in several genes involved in ubiquitination and proteostasis have been identified as causative of IRDs and retinal ciliopathies [16,17,18,19,20].

The DUB enzyme USP48 is expressed in almost all human tissues and, compared to other deubiquitylates, its DUB activity is rather specific, trimming long Lys48-linked ubiquitin chains without completely disassembling them, sparing short ubiquitin chains. The catalytic core of USP48, the ubiquitin C-terminal hydrolase (UCH) domain, is found in its N-terminal end [21,22]. Three DUSP (domain in ubiquitin specific protease) domains, whose function is not well characterised but is associated with protein-protein interaction, follow the UCH domain [23]. At the C-terminus, USP48 presents three serine residues reported to be phosphorylated by casein-kinase-2 (CK2) in the cell nuclei and by glycogen synthase kinase 3β (GSK3β) in the cytoplasm, resulting in the enhancement of USP48 ubiquitin chain-trimming activity [21,24]. At its most C-terminal end, USP48 possesses a ubiquitin-like (UBL) domain that adopts a three-dimensional structure similar to that of ubiquitin and provides a regulatory mechanism of catalytic activity [22,25,26].

USP48 is involved in a wide range of cellular processes such as DNA repair [27,28], cell cycle progression [29], control of immune response [30,31], regulation of the sonic hedgehog pathway [32], and tumorigenesis [23,33,34]. Data on the function of USP48 beyond cancer and its physiological role in other organs is still very scarce [30,35,36,37].

In previous transcriptomic and ChIP-seq analyses performed on mouse retinas, *USP48* showed a differential expression pattern in cone and rod development, being much more expressed in cones, with a strong ChIP-seq peak with CRX, a photoreceptor-specific transcription factor [38]. Furthermore, *Usp48* knockdown in zebrafish resulted in an altered ocular morphology [39]. All these data suggested that USP48 might be important for retinal development and homeostasis.

In this study, we aim to further elucidate the role of USP48 in the retina, particularly in relation to the photoreceptor cilium, and we propose it as a new candidate gene for unsolved retinal ciliopathies.

## 2. Results

### 2.1. USP48 Is Highly Expressed in Cone Photoreceptors

**Figure 1:**
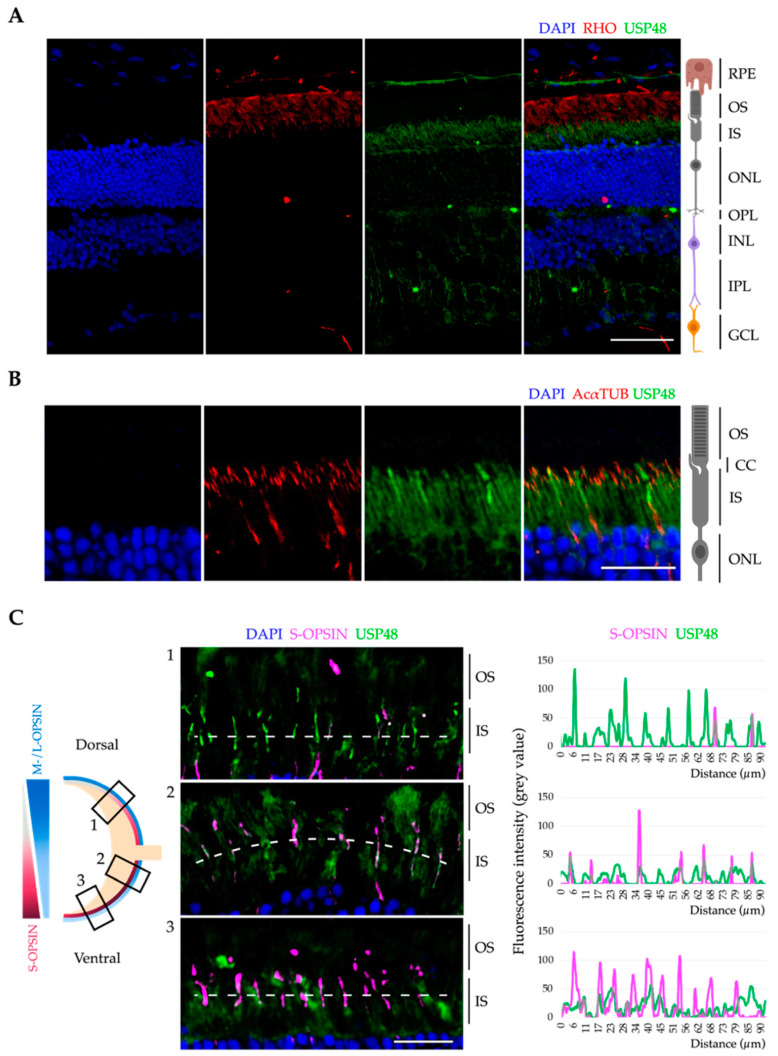
USP48 is expressed in the mouse retina photoreceptors. (**A**) Immunofluorescence on adult mouse retina cryosections shows that USP48 (green) is mainly detected in the IS of photoreceptors and the neuronal plexiform layers. The OS of rod photoreceptors was detected by anti-rhodopsin (RHO, red) and nuclei were counterstained with DAPI (blue). Scale bar: 50 µm. (**B**) Immunofluorescence images of the IS and OS of photoreceptors show that USP48 (green) reaches the base of the CC (highlighted by acetylated α-tubulin (AcαTUB), in red), the bridging structure between the IS and the OS. Nuclei were counterstained with DAPI (blue). The image is one single focal plane. Scale bar: 20 µm. (**C**) At the left, the scheme illustrates the dorso-ventral gradient of M-/L-opsin cones (blue) and S-opsin cones (red) in the mouse retina and the respective position of the immunofluorescence images in the central panel, which reveal that USP48 (green) partially colocalises with S-opsin (magenta). The image is one single focal plane. Scale bar: 20 µm. The right panel depicts the fluorescence intensity plot profile along the discontinuous white line on each image. RPE, retinal pigment epithelium; OS, outer segment; IS, inner segment; ONL, outer nuclear layer; OPL, outer plexiform layer; INL, inner nuclear layer; IPL, inner plexiform layer; GCL, ganglion cell layer; CC, connecting cilium.

First, we assessed the expression of endogenous USP48 in the mouse retina to study its protein levels and localisation along the different neuronal cell layers. Western blotting of protein lysates from the total retina with a commercial antibody against the C-terminal end detected four protein bands, but only three of them had a molecular weight that could be assigned to annotated mRNA isoforms in bioinformatical databases (ENSEMBL and GenomeBrowser-UCSC) (Figure S1). In the retina, USP48 localised mostly at the IS and neuronal plexiform layers (made up of axons and synapses between neurons from adjacent layers), as detected by immunofluorescence on adult mouse retina cryosections (Figure 1A). The highest expression was found at the IS, where USP48 partially colocalises with the CC (Figure 1B).

Some photoreceptors showed higher USP48 fluorescence intensity, reminiscent of the staining usually obtained with cone opsins (Figure 1B). Mouse retinas display an opposite and overlapping distribution pattern of short-wavelength-sensitive opsin (S-opsin) and medium-/long-wavelength-sensitive opsin (M-/L-opsin) cones along the dorso-ventral axis. Thus, the dorsal and ventral regions of the retina can be identified by looking at the expression pattern: S-cones are more concentrated in the ventral retina whereas M-/L-cones are enriched in the dorsal region [40]. To test whether the stronger USP48 fluorescence signal localised with cone photoreceptors, we labelled S-cones on cryosections with a known dorso-ventral orientation. Our results indicate USP48 is expressed in a cone pattern distribution and colocalises with S-opsin in the dorsal and medial retina (Figure 1C).

### 2.2. USP48 Is a Basal Body-Associated Protein

To study endogenous USP48 subcellular and ciliary localisation in a retinal model, we used ARPE-19 and hTERT-RPE1 cells, two human retinal pigment epithelium cell lines, which can develop a cilium when differentiation is induced by serum starvation. 

**Figure 2:**
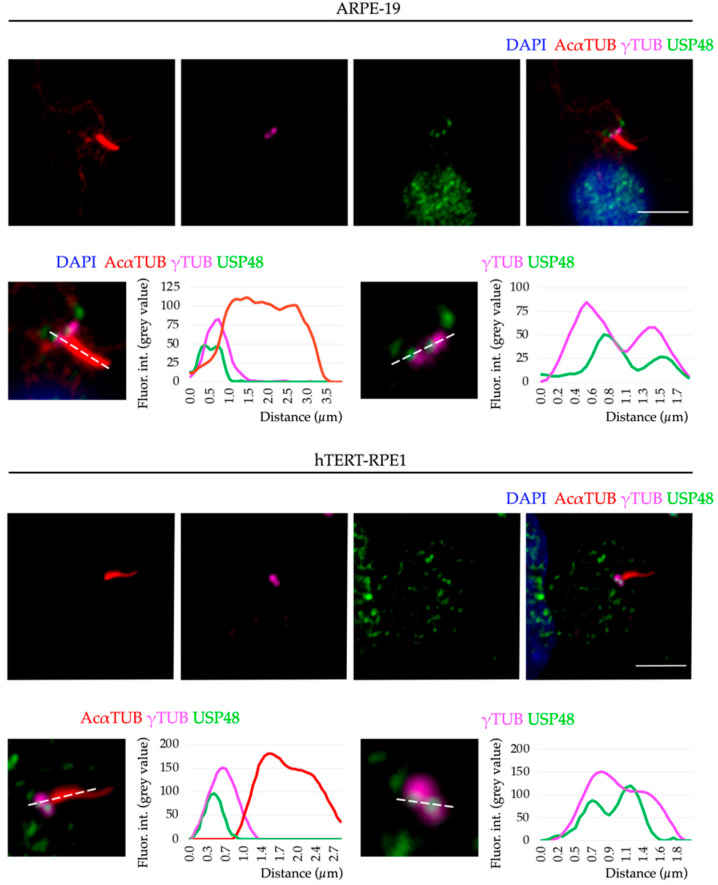
Endogenous USP48 localises to the basal body in ciliated human retinal pigment epithelium cells. Serum-starved ARPE-19 cells (upper panels) and hTERT-RPE1 cells (lower panels) were immunostained with anti-acetylated α-tubulin (AcαTUB, red) to highlight the ciliary axoneme, anti-γ-tubulin (γTUB, magenta) to label the basal body, and anti-USP48 (green). Cell nuclei were counterstained with DAPI (blue). Below each panel, high magnification images of the cilium (left) and basal body (right) with their corresponding plot profiles to quantify the distribution of the fluorescence intensity signal along the discontinuous white lines reveal that a pool of the endogenously expressed USP48 colocalises with γ-tubulin in both human cell lines. The images are one single focal plane. Scale bar: 5 µm. Fluor. int., fluorescence intensity.

In both cell lines, USP48 was mainly localised in the nucleus (as previously reported in replicating cells [23,27,28,41]), but a pool of protein was consistently found at the base of the cilium, the basal body. USP48 localised to both mother and daughter centrioles detected by γ-tubulin, a centrosome marker (Figure 2). The localisation of USP48 to the centrosome is independent of cilium formation, as this specific USP48 localisation was also observed in undifferentiated (non-ciliated) and replicative cells (Figure S2).

**Figure 3:**
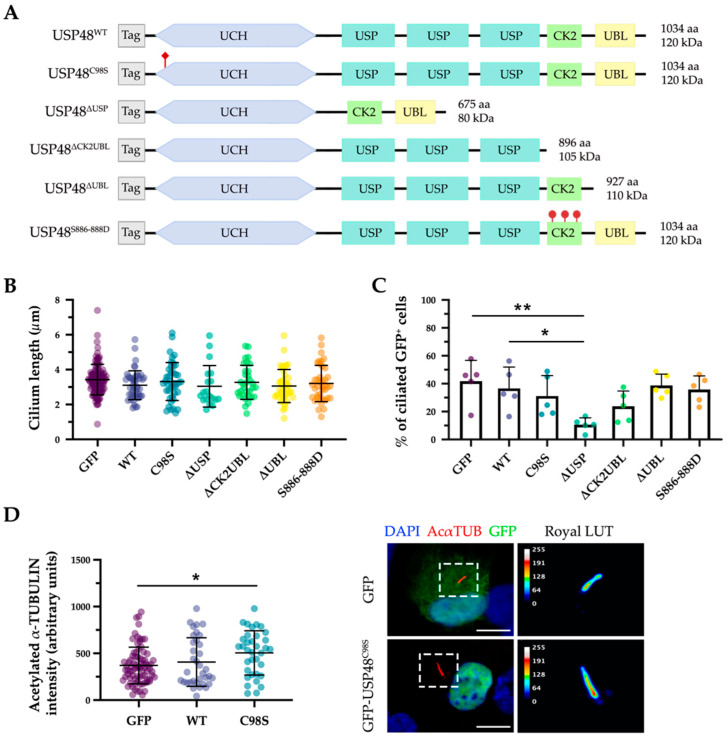
Overexpression of different USP48 mutants results in a mild ciliary phenotype in hTERT-RPE1 cells. (**A**) Schematic representation of the different USP48 mutant constructs used in this study, showing the displayed protein domains, introduced point mutations (rhomboid and round red tags), the amino acid length, and predicted molecular weight of the protein product (excluding the tag of the fusion protein). (**B**) Cilium length of serum-starved hTERT-RPE1 cells transfected with different GFP-USP48 constructs was measured, and an empty GFP vector used as control (one-way ANOVA test; *n* = 20–120; three independent replicates). (**C**) Percentage of ciliated GFP-positive cells was determined by randomly counting >50 cells from each condition, revealing a significant decrease in ciliogenesis when the GFP-USP48^∆USP^ mutant is overexpressed (Kruskal-Wallis test; * = *p*-value ≤ 0.05, ** = *p*-value ≤ 0.01; *n* = 5 independent experiments). (**D**) Quantification of the fluorescence intensity of acetylated α-tubulin in ciliated GFP-positive cells shows a statistically significant increase in cells overexpressing the catalytic mutant GFP-USP48^C98S^ (Kruskal-Wallis test; * = *p*-value ≤ 0.05; *n* = 35–80; three independent experiments). At the right, representative images of the cilium from hTERT-RPE1 cells transfected with GFP (control) and GFP-USP48^C98S^ showing the differences in the fluorescence intensity of acetylated α-tubulin using the ROYAL range of colours (the calibration bar from 0 to 255 grey values is depicted). Cells were immunostained with anti-acetylated α-tubulin (AcαTUB, red) and anti-GFP (green), and nuclei were labelled with DAPI (blue). Data in all the graphics represent the mean ± SD. Scale bar: 10 µm. UCH, ubiquitin C-terminal hydrolase domain; DUSP, domain in ubiquitin-specific protease; CK2, casein-kinase-2 phosphorylation domain; UBL, ubiquitin-like domain; LUT, lookup table.

Next, to assess the role of *USP48* in ciliary formation and function and to identify relevant protein domains, different USP48-derived constructs were used: the wild-type and catalytically inactive mutant (USP48^C98S^), plus several newly generated mutants e.g., three deletion proteins (USP48^∆USP^, USP48^∆CK2UBL^, and USP48^∆UBL^) and one hyper-activated USP48^S886−888D^ (simulating the permanent phosphorylation of the three regulatory serine residues) (Figure 3A).

The ciliary phenotype was analysed on transfected hTERT-RPE1 cells. When overexpressed, neither USP48^WT^ nor any mutant showed any ciliary-compartment localisation (Figure S3). We could not observe differences in the ciliary length in any transfected cells (Figure 3B). However, the overexpression of one of the mutants, GFP-USP48^∆USP^, showed a significant decrease in ciliogenesis, with a lower percentage of ciliated cells in comparison to the control (GFP-transfected cells) and the wild type GFP-USP48 (Figure 3C). On the other hand, overexpression of the catalytically inactive mutant (GFP-USP48^C98S^) resulted in a higher intensity of acetylated α-tubulin in the cilium (Figure 3D).

**Figure 4:**
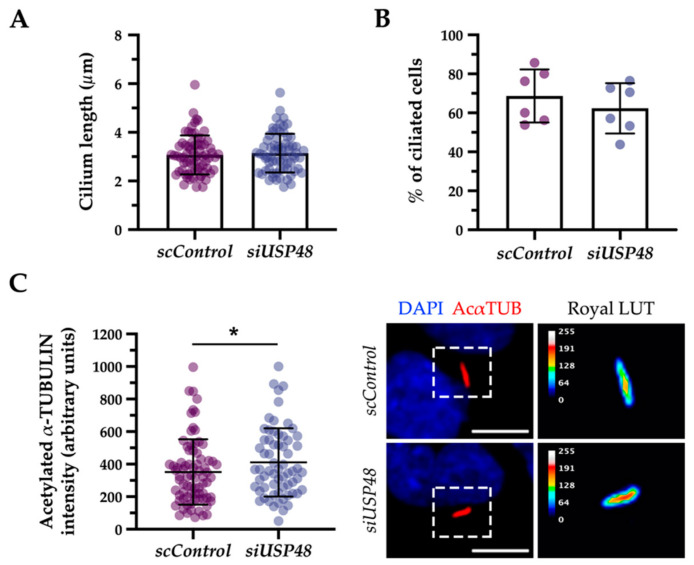
The knock-down of USP48 does not alter ciliogenesis but increases the intensity of ciliary acetylated α-tubulin in hTERT-RPE1 cells. No significant differences were detected in hTERT-RPE1 cells transfected with *scControl* or *siUSP48* with respect to (**A**) the ciliary length (Mann-Whitney test; *n* = 64–69; two independent experiments) and (**B**) the percentage of ciliated cells (Student’s *t*-test; *n* = 3 images with approximately 30 cells/replicate; two independent replicates). (**C**) The fluorescence intensity of the cilium acetylated α-tubulin was significantly increased in cells transfected with *siUSP48* compared to the control (Mann Whitney test; * = *p*-value ≤ 0.05; *n* = 64–78; two independent replicates). At the right, representative images of *scControl*- and *siUSP48*-transfected hTERT-RPE1 cells showing fluorescence intensity differences of acetylated α-tubulin of the cilia using the ROYAL range of colours (the calibration bar from 0 to 255 grey values is depicted). Cells were labelled with anti-acetylated α-tubulin (AcαTUB, red) and nuclei were counterstained with DAPI (blue). All data represent the mean ± SD. Scale bar: 10 µm. LUT, lookup table.

We next assayed the potential effects of silencing *USP48* in ciliogenesis. The knockdown of USP48 by siRNA transfection (silencing efficiency previously tested in [30]), showed similar results to the overexpression experiments: neither ciliary length nor ciliogenesis was clearly affected by USP48 knockdown (Figure 4A,B), although the ciliary acetylate α-tubulin increased (Figure 4C), suggesting that the lack of functional USP48 resulted in hyper-acetylation of α-tubulin in the cilium.

Overall, only the overexpression of some USP48 mutants, or the lack of USP48 DUB activity, altered ciliogenesis or caused a detectable ciliary phenotype, suggesting an indirect role for USP48 in cilium homeostasis.

**Figure 5:**
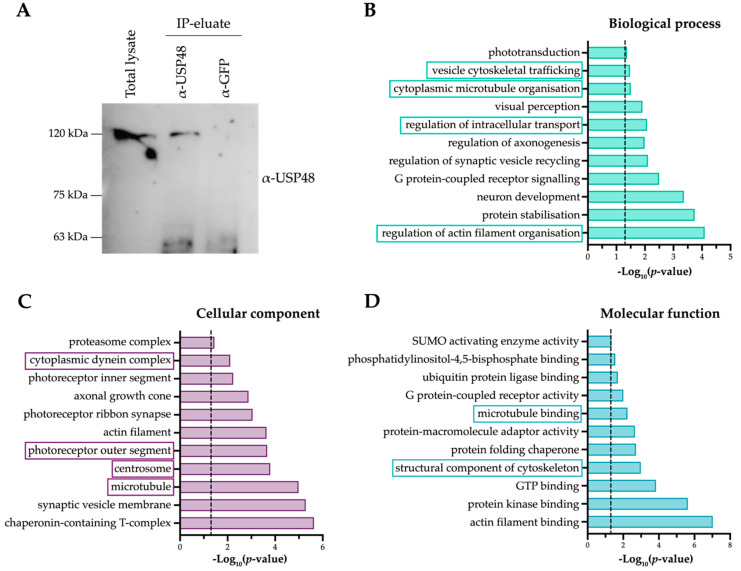
USP48 interacts with proteins involved in ciliary morphogenesis, ciliary trafficking, and photoreceptor-specific functions. (**A**) Endogenous retinal USP48 (approx. 120 kDa) from mouse retinas was successfully and specifically immunoprecipitated in non-denaturing conditions. Anti-GFP was used as a negative control. (**B**–**D**) Graphical representation of the −Log_10_ (*p*-value) of the GO terms of USP48 interactors after proteomics analysis, highlighting specific terms related to ciliary function. Discontinuous lines represent the cut-off of *p*-value ≤ 0.05 in each graphic.

With the aim to further elucidate the role of USP48 in the retina and identify its interactors, we performed a non-denaturing co-immunoprecipitation of mouse retinas with anti-USP48 (Figure 5A). The proteomics results of the eluted proteins by mass spectrometry (Supplementary File S1) were subjected to a Gene Ontology (GO) enrichment analysis to have a functional overview of the principal pathways and cellular subcompartments associated with endogenous USP48 in the retina. As much as 25% of the USP48 interactors were involved in microtubule and actin cytoskeleton and microtubule-dependent intracellular transport, with an enrichment in the “centrosome”, “microtubule”, and “photoreceptor outer segment” terms, which are cilium-related compartments (Figure 5B–D). These protein interactors are consistent with the basal body localisation of the endogenous USP48 (Figure 2) and its high expression in cone OS (Figure 1B,C). “Photoreceptor inner segment” and “photoreceptor ribbon synapse” terms were also enriched, in accordance with USP48 signal in mouse retina cryosections (Figure 1A).

Notably, several interactor proteins that were unambiguously identified are encoded by genes causative of IRDs and/or ciliopathies (Table 1). Worth mentioning interactors are centrosomal proteins CCT2 and rootletin, several dynein chains, and microtubule-associated proteins (e.g., microtubule-associated protein 1B, dynactin 2, cytoplasmic dynein 1 heavy chain 1, cytoplasmic dynein 1 intermediate chain 2, cytoplasmic dynein 1 light intermediate chain), ciliary trafficking proteins (ARL3 and UNC119a) and cargo proteins located at the photoreceptor OS (peripherin, G protein subunit α-transducin 2, guanylate cyclase 2E, ABCA4, or S-opsin). Taken together, our results suggest that USP48 is a basal-body associated protein that may be regulating cilium-related functions in the retina, especially relevant for photoreceptors and human disease.

### 2.3. USP48 Interacts with ARL3 and UNC119a

Among all the identified USP48 interactors, ARL3, and UNC119a were selected for further studies due to their relevance in photoreceptor ciliary transport and involvement in IRDs [42,43].

**Figure 6:**
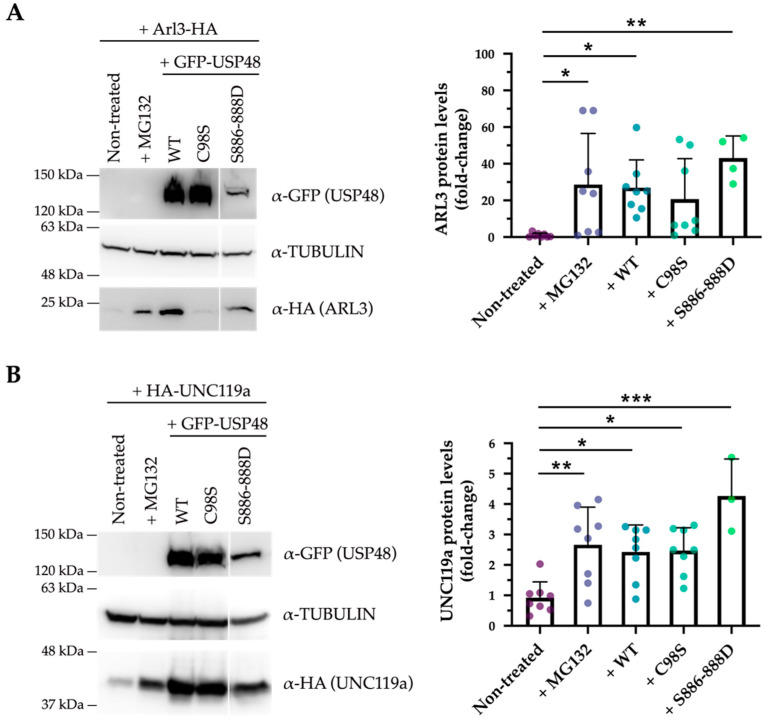
USP48 stabilises ARL3 and UNC119a by different mechanisms. (**A**) ARL3-HA is degraded by the proteasome (rescued by MG-132 treatment) and stabilised by USP48 in a DUB-dependent manner. Note that the hyper-active GFP-USP48^S886−888D^ mutant is able to rescue ARL3 to a higher level (Kruskal-Wallis test; * = *p*-value ≤ 0.05, ** = *p*-value ≤ 0.01; *n* = 4–8 independent replicates). (**B**) HA-UNC119a is also degraded by the proteasome, and the rescue by USP48 is independent of its DUB activity, since the catalytically inactive GFP-USP48^C98S^ mutant is also able to stabilise UNC119a (one-way ANOVA; * = *p*-value ≤ 0.05, ** = *p*-value ≤ 0.01, *** = *p*-value ≤ 0.001; *n* = 3–8 independent experiments). All data represent the mean ± SD.

To study whether the interaction between USP48 and ARL3 and UNC119a was dependent on the USP48 DUB activity, the protein stability of these interactors was assayed in different conditions, and several USP48-derived constructs (USP48^WT^, catalytically inactive USP48^C98S^ or hyperactive USP48^S886−888D^). First, ARL3 and UNC119a showed to be both degraded via the proteasome, as inhibiting the proteasome by adding the proteasome inhibitor MG-132 resulted in a marked increase of the two proteins. Overexpression of USP48 increased the stability of either protein to similar levels to proteasome inhibition (Figure 6). However, the rescue of protein levels was distinct for both interactors. The increase of ARL3 protein stability was dependent on the DUB activity of USP48, as the catalytically inactive USP48^C98S^ mutant could not rescue ARL3 from proteasomal degradation and the hyperactive USP48^S886−888D^ enzyme was significantly more efficient in stabilising ARL3 levels (Figure 6A). On the other hand, UNC119a protein levels were stabilised by all three USP48 variants, irrespective of the DUB activity, indicating that the presence/interaction with the protein was sufficient for UNC119a protein stabilisation (Figure 6B). These results were consistent and independent of the epitope tag used (Figure S4), overall suggesting that the USP48 interaction and stabilisation of ARL3 and UNC119a occur by different mechanisms.

**Figure 7:**
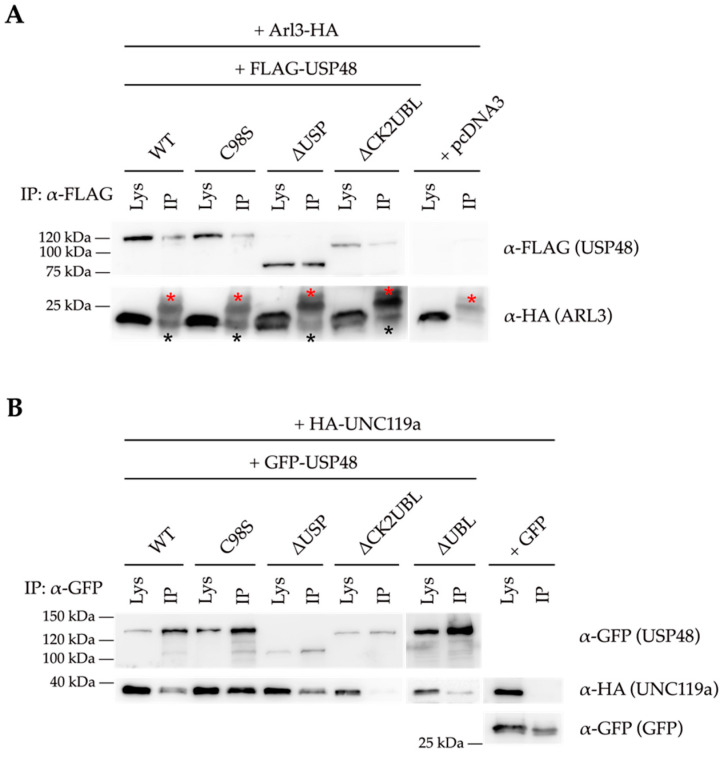
Different domains of USP48 are required for stabilisation of ARL3 and UNC119a. Co-immunoprecipitation using different GFP-USP48 deletion constructs shows that (**A**) the C-terminal domains of USP48 are not required for the interaction with ARL3-HA, and (**B**) the most C-terminal domains (the regulatory CK2 and UBL domains) are responsible for the interaction of USP48 with HA-UNC119a. Note that ARL3 (approx. 23 kDa, indicated by a black asterisk) has a similar molecular weight to the light IgG chains (red asterisk). Lys, lysate; IP, immunoprecipitate.

In order to dissect which USP48 domain was involved in the interaction, we performed co-immunoprecipitation experiments with either ARL3 or UNC119a and several USP48 proteins carrying different domain deletions. ARL3 co-immunoprecipitated with all USP48-derived proteins, indicating that the interaction between USP48 and ARL3 most probably occurs via the UCH catalytic domain of USP48 (Figure 7A). On the other hand, UNC119a did not co-immunoprecipitate with the USP48^∆CK2UBL^ mutant, in which the C-terminus of USP48 (spanning the CK2 and UBL regulatory domains) has been deleted, and showed a lower affinity for the USP48^∆UBL^ in comparison with the other protein constructs (Figure 7B). This result suggested that the CK2 domain, and to a minor extent the UBL domain, were crucial for the interaction between UNC119a and USP48.

In summary, all our data support that USP48 can interact with and stabilise ARL3 and UNC119a using different mechanisms and distinct protein domains.

## 3. Discussion

An increasing number of reports about the role of the ubiquitin-proteasome system in ciliogenesis, retina development, and homeostasis have been published of late, thus hinting at the importance of proteostasis in these finely-tuned pathways [44,45,46,47]. As such, mutations in genes encoding DUB enzymes have been identified as causative of IRDs and ciliopathies (with or without retinal involvement) [16,17,18,20]. However, as happens with other rare diseases, there are still patients without a positive genetic diagnosis and the waiting time to reach diagnosis can exceed half a decade [48]. Many unsolved cases require the identification of novel causative genes and mutations.

We here demonstrate that USP48 is expressed in the retina, especially at cone photoreceptors as supported by immunofluorescence on mouse retina cryosections and proteomics, which identified cone-specific proteins as USP48 interactors (among others, S-opsin, phosphodiesterase 6C, and G protein subunit α-transducin 2). These results are in complete agreement with previous RNA-seq and ChIP-seq analyses which detected a strong *Usp48* expression in the retina, particularly in flow-sorted cones [38]. Interestingly, in previous reports using cancer models and replicative cells, USP48 was usually localised at the cell nucleus [23,41,49] but it may have a more subtle subcellular localisation in differentiated neurons, localising to synaptic sites and perinuclear cytosol [35,36]. These changes in USP48 subcellular localisation may underlay neuron-specific functions. In fact, neural tissues present the highest number of alternative splicing events among all tissues, and photoreceptors have an even more complex retina-specific splicing program, reflecting the highly specialised architecture and function of retinal cells [50]. Some examples are *RPGR* and *BBS8*, both causative of non-syndromic retinal ciliopathies when their mutations map in retina-specific exons exclusively present in photoreceptor isoforms [51,52]. Interestingly, a recent study showed that retina-enriched microexons are particularly present in genes involved in vesicle-mediated transport, cilium assembly, and ciliary trafficking [53]. USP48 has many annotated isoforms, but only the full-length protein has been studied so far. In cultured epithelium retinal cells, most USP48 localises at the nucleus, but we here demonstrate for the first time that an endogenous USP48 protein that at least contains the C-terminus (where the epitope of the used antibody is located) localises to the basal body and centrosome, in either differentiated or replicative stages. However, the overexpressed full-length USP48 protein was not detected at the base of the cilium, suggesting that this basal body/ciliary-related localisation might be restricted to a particular protein isoform—most probably retina-specific—since the localisation and protein interactome of the endogenous USP48 in photoreceptors also indicates its association with the basal body and connecting cilium. Further work is needed to confirm the presence of this putative isoform.

Based on our data from USP48 overexpression and siRNA knockdown assays, USP48 is most probably a non-essential component of the centrosome, as no strong ciliary defects were observed in these conditions. However, several signs of ciliogenesis impairment after overexpression of the USP48^∆USP^ mutant suggest that, while USP48 lack of function does not perturb cilium formation, some USP48 mutant proteins might act as inactive proteins that hijack cilium-associated interactors, resulting in a dominant-negative effect. Therefore, even if USP48 is dispensable for ciliogenesis in retinal pigment epithelium cells, it probably acts as a modulator by fine-tuning cilium interaction networks, which might be more sensitive to the lack of USP48 in tightly regulated contexts, as the photoreceptor. In fact, the results obtained when analysing the ciliary acetylated α-tubulin fluorescence intensity support a potential regulatory role for USP48. Tubulin acetylation increases microtubule flexibility, protecting microtubules from mechanical stress and rendering them more stable. As a consequence, acetylated microtubules exhibit an enhanced protein trafficking [54,55]. In the *siUSP48*- and USP48^C98S^-transfected conditions, where USP48 activity is lost or strongly depleted, cilium axonemes displayed higher levels of α-tubulin acetylation compared to controls. In line with these results, among the USP48 interactors in retinal lysates, we detected microtubule-associated protein MAP1B (inducer of microtubule acetylation in neurons) as well as many other MAPs (microtubule-associated proteins) [56].

Protein transport to the OS is crucial for photoreceptor homeostasis due to the continuous shedding of the membranous disks, hence the need for a properly operating ciliary trafficking machinery [57]. We selected ARL3 and UNC119a, among USP48 interactors in the retinal proteome, for further work due to several reasons: (1) mutations in *UNC119a* and *ARL3* cause retinal dystrophy and, in the case of the latter, also a syndromic ciliopathy; and (2) both proteins are involved in the delivery of lipidated proteins to the photoreceptor OS, a pathway known as lipidated protein intraflagellar transport (LIFT). In fact, these two proteins also interact as UNC119a is a chaperone that transports cargo to the OS where it associates with ARL3 for cargo release. This interaction enables the translocation of both proteins back to the IS, where they dissociate to start the transport cycle again [42,43]. In addition, UNC119a cargo proteins (e.g., cone-type 11transducin α-subunit) were also identified in the proteomic analyses.

Our data show that USP48 stabilises the levels of ARL3 and UNC119a using distinct mechanisms: while ARL3 stabilisation seems to be dependent on USP48 DUB activity, the stabilisation of UNC119a by USP48 is unrelated to its DUB activity. The ubiquitin-specific protease (USP) family proteins are large polypeptides with many domains that can exert functions beyond the DUB catalytic activity, and non-canonical and DUB-independent mechanisms of protein level regulation have been reported for USP48 as well as other USPs [58,59]. For instance, USP48 might bind UNC119a and block the recognition site of the E3 ligase, preventing UNC119a from being ubiquitinated and targeted for proteasomal degradation. Alternatively, the DUB-independent regulation of UNC119a levels by USP48 could be hinting at an indirect interaction between UNC119a and USP48, through a third partner or via the formation of a stabilising complex with USP48, resulting in the stabilisation of UNC119a. The differential mechanism of stabilisation of ARL3 and UNC119a is further supported by their interaction with USP48 via distinct domains. After using several USP48 deletion mutants, we inferred that ARL3 and USP48 interact through the UCH domain of USP48, irrespective of the catalytic activity (as described with other USP48 substrates [32]), whereas USP48 interacts with UNC119a through its most C-terminal region, where the CK2 and UBL regulatory domains are located. Indeed, evidence of the crucial role of ubiquitination and other post-translational modifications in the regulation of ciliary transport is increasing [60]. For instance, the ubiquitin ligase CUL3-KLHL18 was described to regulate LIFT via UNC119a ubiquitination, thereby promoting its proteasomal degradation [61]. Our results suggest that USP48 might also participate in the regulation of LIFT in photoreceptors.

Moreover, UNC119a is involved in neuron polarity by organising microtubule cytoskeleton [62] and ARL3 participates in the regulation of dynactin-bound cargo dissociation from dynein motors [63]; both functions are also compatible with the obtained retinal USP48 proteome as many interactors are related to cytoskeleton organisation and motor-mediated intracellular transport (e.g., tubulin α-4A, microtubule-associated proteins 1B, 2, 4 and 6, dynactin 1, dynactin 2, cytoplasmic dynein 1 heavy chain 1, cytoplasmic dynein 1 intermediate chain 2, cytoplasmic dynein 1 light intermediate chain 1). Of note, in addition to their ciliary localisation, both ARL3 and UNC119a are found at the synaptic ribbon complex in photoreceptors, where they have also been involved in the transport of lipid-modified proteins to ribbon synapses [64,65]. In agreement with the observed localisation of USP48 at synaptic sites [36] and plexiform layers in our immunostained retinal cryosections, USP48 might also be modulating the functions of UNC119a and ARL3, not only in the OS and cilium but in synapses too.

Finally, over the past few years, there has been a growing interest in the development of small-molecule DUB inhibitors as therapeutic agents, mainly to treat cancer [66]. Our results indicate that USP48 inhibition might be a good therapeutic strategy to counteract the effect of *ARL3* variants causing autosomal dominant RP [67,68], but further research is needed since, to the best of our knowledge, no USP48-specific inhibitor has been developed so far.

In summary, USP48 interacts with many cytoskeletal and cilium-associated proteins and its functions probably range from the modulation of the ciliary and synaptic transport to the modulation of cytoskeleton organisation, which are key for photoreceptor function and homeostasis. Indeed, variants in USP48 have been associated with autosomal dominant non-syndromic hereditary hearing loss [35] further supporting a critical role in neural, neurosensory, and other ciliated tissues. Additional work is needed to shed light on the role of USP48 in the retina, but our results posit it as a new candidate/modifier gene for unsolved ciliopathies and inherited retinal dystrophies.

## 4. Materials and Methods

### 4.1. Animal Handling

Tissue from WT C57BL6/J mice was obtained according to the ARVO statement for the use of animals in ophthalmic and vision research under the regulations of the Ethical Committee for Animal Experimentation (AEC) of the Generalitat of Catalonia according to the European Directive 2010/63/EU and other pertinent national laws.

### 4.2. Cell Culture and Transfections

Human hTERT-RPE1 cells (from Garcia-Gonzalo’s lab) and ARPE-19 cells (ATCC, CRL_2302) were cultured in 10% foetal bovine serum (FBS) (Life Technologies, Carlsbad, CA, USA) and 1% penicillin/streptomycin (Life Technologies, Carlsbad, CA, USA) in 1:1 Dulbecco’s Modified Eagle’s Medium (DMEM) (ATCC, Manassas, VA, USA) and Ham’s F-12 Nutrient Mix (F12) (Life Technologies, Carlsbad, CA, USA) in a 5% CO_2_ cell culture humidified incubator at 37 °C. To induce differentiation, hTERT-RPE1 cells were incubated with 0.2% FBS for 24 h and ARPE-19 cells with no FBS for 48 h.

For plasmid reverse transfection, cells in suspension (2 × 10^5^ cells/well on coverslips in 24-well plates) were transfected using Lipofectamine 2000 (Invitrogen, Carlsbad, CA, USA) (DNA–Lipofectamine ratio 1:2) in non-antibiotic medium, and 4 h post-transfection, the non-antibiotic medium was replaced with fresh complete medium, which was then replaced with differentiation medium, 24 h after transfection.

siRNA transfection was performed by transfecting cells in suspension (2 × 10^5^ cells/well on coverslips in 24-well plates) with 1 µL lipofectamine RNAiMAX reagent (Thermo Fisher Scientific, Rockford, IL, USA) and 10 nM non-targeting siRNA (*siControl*) (Dharmacon, Lafayette, CO; D-001810-01-05) or anti-USP48 small interfering RNA (*siUSP48*) (Dharmacon, Lafayette, CO; J-006079-11-0002). Cells were also serum-starved 24 h after transfection.

For Western blotting experiments, human HEK293 cells were grown in Dulbecco’s Modified Eagle’s Medium (DMEM) (ATCC, Manassas, VA, USA) supplemented with 10% Foetal Bovine Serum (FBS) (Life Technologies, Carlsbad, CA, USA) and 1% Penicillin- Streptomycin (Life Technologies, Carlsbad, CA) in a 5% CO_2_ cell culture humidified incubator at 37 °C. Prior to transfection, HEK293 cells were seeded onto 12-well plates (2.5 × 10^5^ cells per well) or 6-well plates (5 × 10^5^ cells per well) and incubated for 24 h. Plasmids (0.75–3 µg of total DNA) were transfected in the non-antibiotic medium using Lipotransfectine (Niborlab, Guillena, Spain) (DNA–lipotransfectine ratio 1:2). After 5 h, the non-antibiotic medium was replaced with complete medium for 48 h. For protein stability analysis, 32 h after transfection cells were treated with 10 µM MG-132 (Sigma-Aldrich, St. Louis, MO, USA; 474790) for 16 h.

### 4.3. Plasmid Vectors and Plasmid Constructions

pcDNA3-FLAG-USP48 and pcDNA3-FLAG-USP48^C98S^ were kind gifts from Dr. George Mosialos [31]. pcDNA6.2-emGFP-USP48 and pcDNA6.2-emGFP-USP48^C98S^ expression constructs were generously provided by Dr. Joanna I. Loizou [27].

pcDNA3-Arl3-6xHis, pcDNA3-Arl3-HA and pcDNA3-HA-UNC119a were cloned from pET20b-Arl3-6xHis and pGEX-4T-GST-UNC119a plasmids, kind gifts from Dr. Shehab Ismail [69], to pcDNA3 and pcDNA3-HA vectors. Arl3-6xHis insert was obtained from PCR amplification with a BamHI restriction site-containing forward primer: 5′ GGGGATCCATGGGCTTGCTCTCTATTTTG 3′ and an EcoRI target site-containing reverse primer: 5′ GGAATTCTCAGTGGTGGTGGTGGTG 3′. Afterward, both insert and vectors pcDNA3 were digested with FastDigest BamHI and FastDigest EcoRI (Thermo Fisher Scientific, Rockford, IL, USA; FD0054 and FD0274, respectively) enzymes, followed by ligation. The pcDNA3-Arl3-HA plasmid was generated by amplifying Arl3 using the primers 5′ CCCAAGCTTATGGGCTTGCTCTCTATTTTG 3′ and 5′ CGGGATCCGTTTCTTCTTTGCGTTGACATTC 3′, respectively including a HindIII and a BamHI restriction sequences. Then, both the insert and the pcDNA3-HA plasmid were digested with FastDigest BamHI and FastDigest HindIII (Thermo Fisher Scientific, Rockford, IL, USA; FD0054 and FD0504, respectively) for cassette exchange. UNC119a was amplified by PCR from pGEX-4T-GST-UNC119a using the forward primer 5′ TCCCCGCGGCCATGAAGGTGAAGAAGGG 3′, which inserted a SacII target sequence, and the reverse primer 5′ GTCAGTCAGTCACGATGCG 3′. Next, the pcDNA3-HA destination plasmid and the amplicon were digested with SacII (Promega, Madison, WI, USA; R622A) and FastDigest EcoRI (Thermo Fisher Scientific, Rockford, IL, USA; FD0274) and ligated.

To obtain the different USP48 mutants, distinct strategies were followed using the pcDNA3-FLAG-USP48 vector as a starting point. pcDNA3-emGFP-USP48^ΔUSP^ (c.[1378_2460del;1377_1378insGAATTC], p.[Q460_R820del; K459_I460insEF]) was constructed by inverse PCR using the forward primer 5′ GGAATTCATTGAAGTGGGAGATGTAAACC 3′ and the reverser primer 5′ GGAATTCCTTACGCATCTCAGCCATTTC 3′, both including an EcoRI restriction site at their 5′ end to facilitate enzymatic ligation after restriction digestion. To generate the pcDNA3-emGFP-USP48^ΔCK2UBL^ mutant (c.26342_3105del; p.E881VfsX16), the pcDNA3-FLAG-USP48 plasmid was digested with FastDigest BspEI/Kpn2I and FastDigest XhoI (Thermo Fisher Scientific, Rockford, IL, USA; ER0531 and ER0531, respectively) restriction enzymes. Next, 1 unit/DNA µg of Klenow Polymerase I Large Fragment (Promega, Madison, WI, USA; M220A) was used to fill up the blunt ends for 30 min at 37 °C, followed by ligation. In the pcDNA3-emGFP-USP48^ΔUBL^ construct (c.2781_2782insT, p.K928X), a single nucleotide insertion upstream the UBL domain was inserted by inverse PCR with the primers 5′ CCCATCAAAATTATATAGCCTATCAATAAGCAAGTTATTCGCC 3′ and 5′ GGCGAATAACTTGCTTATTGATAGGCTATATAATTTTGATGGG 3′ to generate a frameshift mutation and, consequently, a premature STOP codon. DpnI (Promega, Madison, WI, USA; R6231) digestion for 1 h at 37 °C was performed to eliminate the original vector and increase the efficiency yield of mutant construct obtention. Finally, the pcDNA3-emGFP-USP48^S886−888D^ mutant (c.2653_2660AGTAGTTC>GATGATGA, p.S886_888D) was obtained by site-directed mutagenesis of the two first nucleotides of each codon by a two-step PCR strategy. First, a 570 bp fragment was amplified using a forward primer that introduced the desired mutation and included at its 5′ end a Kpn2I restriction site: 5′ GAAGGATTCGGCTCCGGAACTGAATGTGGATGATGATGAAACAGAGGAGGACAAGGAAGAAGCT 3′, and a reverse primer that contained an XhoI targe site at its 5′ end: 5′ CTCTAGATGCATGCTCGAGCG 3′. Afterward, a cassette exchange with the original pcDNA3-FLAG-USP48 using FastDigest BspEI/Kpn2I and FastDigest XhoI (Thermo Fisher Scientific, Rockford, IL, USA; ER0531 and ER0531, respectively) restriction enzymes was performed. Once the pcDNA3-FLAG-USP48 mutants were obtained, the FLAG tag was replaced by the emGFP tag from the pcDNA6.2-emGFP-USP48 plasmid. First, emGFP was amplified by PCR on pcDNA6.2-emGFP-USP48 plasmid using the forward primer 5′ GGGGTACCATGGTGAGCAAGGGC 3′, which incorporated a KpnI restriction site, and the reverse primer 5′ GCTCCTGCGACACCTCCTC 3′, which included a SacII target site. Then, both the amplified emGFP PCR product and the destination plasmids (pcDNA3-FLAG-USP48 mutants) were digested with FastDigest KpnI (Thermo Fisher Scientific, Rockford, IL, USA; FD0528) and SacII (Promega, Madison, WI, USA; R622A) and ligated.

All PCR were performed using the AccuPrime^TM^ Taq DNA Polymerase, High Fidelity (Thermo Fisher Scientific, Rockford, IL, USA; 12346086) followed by purification of PCR products or DNA band from agarose gel with the NZYGelpure Kit (NZYTech–Genes&Enzymes, Lisbon, Portugal; MB01101), all according to the manufacturer’s protocol. Enzymatic restrictions were carried out for 1–2 h at 37 °C and enzymes were inactivated at 80 °C for 5 min. Finally, all ligations were performed using T4 DNA ligase (Thermo Fisher Scientific, Rockford, IL, USA; EL0011) for 6 h at room temperature or overnight at 17 °C, and the ligation product was transformed into DH5α *E. coli* bacteria (Thermo Fisher Scientific, Rockford, IL, USA; 18265017).

### 4.4. Immunohistochemistry on Mouse Retinal Cryosections

Briefly, eyes from adult mice were enucleated, fixed in 4% paraformaldehyde (PFA), and embedded in OCT [70]. Twelve μm retinal sections were recovered on commercial Superfrost Plus glass slides (Electron Microscopy Sciences, Hatfield, PA) and kept frozen at −80 °C until use. Cryosections were rehydrated with phosphate-buffered saline (PBS) and permeabilised in 0.5% Triton X-100 (Sigma-Aldrich, St. Louis, MO, USA) in PBS for 20 min. Sections were then subjected to antigen retrieval using 0.05 mg/mL Proteinase K (Sigma-Aldrich, St. Louis, MO, USA; P2308) in PBS for 2 min and blocked in 5% sheep serum (Merck, Darmstadt, Germany; S22) in PBS for 1 h. Incubations with the primary antibodies anti-USP48 (Abcam, Plc, Cambridge, UK; ab72226; 1:50), anti-RHODOPSIN (Abcam, Plc, Cambridge, UK; ab5417; 1:250), anti-acetylated α-TUBULIN (Sigma-Aldrich, St. Louis, MO, USA; T6793; 1:1000) and anti-S-OPSIN (Thermo Fisher Scientific, Rockford, IL, USA; 600-101-MP7; 1:50) were performed overnight at 4 °C. After three washes with PBS, cryosections were incubated in a blocking solution for 1 h at room temperature with the corresponding secondary antibodies: AlexaFluor 568 anti-Mouse (Thermo Fisher Scientific, Rockford, IL, USA; A11004; 1:400), AlexaFluor 488 anti-Rabbit (Thermo Fisher Scientific, Rockford, IL, USA; A11070; 1:400), and AlexaFluor 633 anti-Goat (Thermo Fisher Scientific, Rockford, IL, USA; A21082; 1:100), and nuclei were stained with 4′,6-diamidine-2′-phenylindole dihydrochloride (DAPI) (Sigma-Aldrich, St. Louis, MO, USA; 10236276001; 1:1000). Finally, the slides were rinsed with PBS and coverslipped with ProLong™ Gold Antifade Mountant (ThermoFisher Scientific, Rockford, IL, USA; P1014).

### 4.5. Immunocytochemistry

hTERT-RPE1 cells were fixed in 2% PFA for 5 min followed by 4% PFA for 30 min at room temperature. Afterward, cells were washed in PBS, permeabilised in 0.5% Triton X-100 (St. Louis, MO, USA) in PBS for 20 min at room temperature, and blocked for 1 h in either 3% bovine serum albumin (BSA) (NZYTech, Lisbon, Portugal; MB04602) or in 10% Normal Goat Serum (NGS) (Thermo Fisher Scientific, Rockford, IL, USA; 16210064) in PBS with 0.2% Triton X-100 (St. Louis, MO, USA). The following primary antibodies were incubated overnight at 4 °C in blocking solution: anti-USP48 (Abcam, Plc, Cambridge, UK; ab72226; 1:50), anti-γ-TUBULIN (Sigma-Aldrich, St. Louis, MO, USA; T6557; 1:500), anti-GFP (Abcam, Plc, Cambridge, UK; ab290; 1:500), anti-HA (BioLegend, San Diego, CA, USA; 901502; 1:250), and anti-FLAG (BioLegend, San Diego, CA, USA; 637301; 1:250). After incubation, cells were rinsed with PBS and incubated with the corresponding secondary antibodies in a blocking solution for 1 h at room temperature: AlexaFluor 568 anti-Mouse (Thermo Fisher Scientific, Rockford, IL, USA; A11004; 1:300), AlexaFluor 488 anti-Rabbit (Thermo Fisher Scientific, Rockford, IL, USA; A11070; 1:300), AlexaFluor 647 anti-Mouse (Thermo Fisher Scientific, Rockford, IL, USA; A21235; 1:300) AlexaFluor 488 anti-Rat (Thermo Fisher Scientific, Rockford, IL, USA; A11006; 1:300). Nuclei were stained with 4′,6-diamidine-2′-phenylindole dihydrochloride (DAPI) (Sigma-Aldrich, St. Louis, MO, USA; 10236276001; 1:1000). A 2-h incubation at room temperature was used for AlexaFluor 546-conjuaged acetylated α-TUBULIN (Santa Cruz Biotechnology, Dallas, TX, USA; sc-23950 AF546; 1:250). Finally, coverslips were washed with PBS and mounted using Mowiol 4-88 (Merck, Kenilworth, NJ, USA).

### 4.6. Microscope Image Acquisition and Analysis

Samples were analysed by confocal microscopy (Zeiss LSM 880, Thornwood, NY, USA) and images were collected using ZEN-LSM software. The ImageJ software (National Institutes of Health, Bethesda, MD) was used for processing, measurement, and analyses of confocal immunofluorescence images. Fluorescence intensity plot profiles were generated from one representative immunofluorescence image per condition. The ROYAL lookup table (LUT) was employed to transform the intensity grey values (from 0 to 255) to coloured pixels and facilitate the visualisation of differences in fluorescence intensity between the representative immunofluorescence images of each condition. Acetylated α-tubulin fluorescence intensity was measured using the integrated density parameter in the ImageJ software (National Institutes of Health, Bethesda, MD, USA).

Cilia length was measured from the base of the cilium (immunostained with anti-γ-tubulin) to the tip of the axoneme (labelled with anti-acetylated α-tubulin). For quantitative analysis of ciliogenesis, the percentage of ciliated cells versus non-ciliated cells was counted manually, considering all the cells in the field in the siRNA transfection experiments, or only the GFP-positive cells in the GFP-USP48 overexpression assays.

### 4.7. Co-Immunoprecipitation Assays

Retinas from WT mice as well as transfected HEK293 cells were resuspended in RIPA lysis buffer (50 mM TrisHCl pH 7.5, 1 mM EDTA, 150 mM NaCl 0.5% Nonidet P-40) and protease inhibitor cocktail (Complete, Roche Diagnostics, Indianapolis, IN) and incubated 30 min on ice. Protein extracts were recovered after centrifugation and, after saving 10% of the supernatant (input), lysates were incubated overnight at 4 °C in rotation with the corresponding primary antibody (anti-USP48 (Abcam, Plc, Cambridge, UK; ab72226; 3 µg antibody/mg protein) and anti-GFP (Abcam, Plc, Cambridge, UK; ab290; 3 µg antibody/mg protein) for mouse retinal extracts, or anti-HA (BioLegend, San Diego, CA; 901502; 1:100), anti-FLAG (Thermo Fisher Scientific, Rockford, IL, USA; PA1-984B; 1:80), and anti-GFP (Abcam, Plc, Cambridge, UK; ab290; 1:100) for overexpression experiments in HEK293 cells. Protein G Sepharose 4 Fast Flow beads (Merck, Darmstadt, Germany; GE17-0618-01) were washed with RIPA lysis buffer three times and the overnight antibody-bound extracts were added to the pelleted beads and incubated in a rotating wheel for 4 h at room temperature. After three rounds of centrifugation (30 s at 3000 rpm) and RIPA lysis buffer rinsing, bound proteins were eluted from the beads by boiling 5 min with protein loading buffer 2× (120 mM TrisHCl pH 6.8, 20% glycerol, 4% SDS, 0.2% bromophenol blue and 10% β-mercaptoethanol).

### 4.8. Western Blot

Retina lysates from WT mice were obtained by homogenisation in RIPA lysis buffer (50 mM TrisHCl pH 7.5, 1 mM EDTA, 150 mM NaCl, 0.25% Sodium Deoxycholate) containing cOmplete^TM^ Mini protease inhibitors (Roche Diagnostics, Indianapolis, IN, USA), centrifugation and subsequent supernatant collection. Cells were lysed in SDS-PAGE protein loading buffer 1x (60 mM TrisHCl pH 6.8, 10% glycerol, 2% SDS, 0.1% bromophenol blue and 5% β-mercaptoethanol).

Proteins were analysed by 10% or 12% SDS-PAGE and transferred onto PVDF membranes (BioRad, Hercules, CA, USA; 1620177), blocked with 10% non-fat milk or 3% BSA (NZYTech, Lisbon, Portugal; MB04602) in PBS containing 0.1% Tween 20 (Sigma-Aldrich, St. Louis, MO, USA; P2287) for 1 h and incubated overnight at 4 °C with primary antibodies: anti-USP48 (Abcam, Plc, Cambridge, UK; ab72226; 1:1000), anti-α-TUBULIN (Sigma-Aldrich, St. Louis, MO, USA; T5168; 1:1000), anti-GFP (Abcam, Plc, Cambridge, UK; ab5417; 1:1000), anti-FLAG (Thermo Fisher Scientific, Rockford, IL, USA; PA1-984B; 1:500), anti-6xHis (BioLegend, San Diego, CA, USA; 906113; 1:1000), and anti-HA (BioLegend, San Diego, CA, USA; 901502; 1:1000). After incubation with horseradish peroxidase-conjugated secondary antibodies (1:2000) for 1 h at room temperature, membranes were revealed with the ECL^TM^ Prime Western Blotting System (Sigma-Aldrich, St. Louis, MO, USA; GERPN2236). Images were acquired by ImageQuant™ LAS-4000 mini Image Analyzer (Fujifilm, Tokyo, Japan) and the ImageJ software (National Institutes of Health, Bethesda, MD, USA) was used for quantification using α-tubulin density as a loading control.

### 4.9. Mass Spectrometry and Proteomic Analysis

Twelve retinas from 3 months-old mice were dissected, disrupted with a tissue homogeniser for immunoprecipitation using the protocol detailed in Section 4.7, and incubated with anti-USP48 (Abcam, Plc, Cambridge, UK; ab72226; 3 µg antibody/mg protein) and anti-GFP (Abcam, Plc, Cambridge, UK; ab5417; 3 µg antibody/mg protein) as negative control. Samples eluted from the Protein G Sepharose 4 Fast Flow beads were separated in 10% SDS-PAGE and entire gel lanes were excised.

Samples were processed and analysed at the Proteomic facility of PCB (Proteomics unit, Parc Científic de Barcelona, Barcelona, Spain). Proteins present in the gel bands were fixed and in-gel digested with trypsin (Promega, Madison, WI, USA; V5111) at 37 °C, pH 8. The resulting peptide mixtures were extracted from the gel matrix and cleaned up with a C18 tip (PolyLC Inc, Columbia, MD, USA; TT10C18) as per the manufacturer’s protocol. Finally, the cleaned-up peptide solutions were dried-down in a SpeedVac vacuum system (Thermo Fisher Scientific, Rockford, IL, USA) and stored at −20 °C until liquid chromatography-mass spectrometry (LCMS) analyses. The dried-down peptide mixtures were analysed in a nanoACQUITY liquid chromatographer (Waters, Milford, MA, USA) coupled to an LTQ-Orbitrap Velos (Thermo Fisher Scientific, Rockford, IL, USA) mass spectrometer. The tryptic digests were resuspended in 1% formic acid solution and corresponding aliquots were injected for chromatographic separation. Peptides were trapped on a ACQUITY Symmetry C18^TM^ trap column (5 µm × 180 µm × 20 mm; Waters, Milford, MA, USA), and were separated using the C18 reverse phase capillary column ACQUITY UPLC BEH column (130 Å, 1.7 µm, 75 µm × 250 mm; Waters, Milford, MA, USA). Eluted peptides were subjected to electrospray ionisation in an emitter needle (PicoTip^TM^, New Objective, Zurich, Switzerland) with an applied voltage of 2000 V. Peptide masses (300–1600 *m*/*z*) were analysed in a data-dependent mode where a full Scan MS was acquired in the Orbitrap with a resolution of 60,000 FWHM at 400 *m*/*z*. Up to the 15th, most abundant peptides (minimum intensity of 500 counts) were selected from each MS scan and then fragmented in the linear ion trap using CID (38% normalised collision energy) with helium as the collision gas. The generated raw data files were collected with Thermo Xcalibur (v.2.2, Thermo Fisher Scientific, Rockford, IL, USA).

A database was created by all protein entries for *Mus musculus* present in the database SWISS-PROT [71]. A small database containing laboratory contaminant proteins was also added. The raw files obtained in the mass spectrometry analyses were used to search this database using the SEQUEST HT search engine [72] with the Proteome Discover^TM^ software (v.1.4.1.14, Thermo Fisher Scientific, Rockford, IL, USA). To improve the sensitivity of the database search, Percolator (semi-supervised learning machine) was used in order to discriminate correct from incorrect peptide spectrum matches. The percolator assigns a q-value to each spectrum, which is defined as the minimal FDR at which the identification is deemed correct. These q-values are estimated using the distribution of scores from the decoy database search. Finally, only proteins identified with at least 2 peptides (FDR ≤ 5%) were considered for further analysis. Proteins also present in the anti-GFP sample were discarded. Gene Ontology (GO) enrichment analysis was performed using the web-tools DAVID 2021 (DAVID Bioinformatic Database, Frederick, MD, USA) [73,74] and PANTHER classification system v.17.0 (PANTHER Classification System, Los Angeles, CA, USA) [75,76].

### 4.10. Statistical Analyses

Data were analysed using GraphPad Prism software (GraphPad v9.0.1 Software Inc., San Diego, CA, USA). Homoscedasticity and normality were verified using Bartlett’s test, and D’Agostino and Person’s test, respectively. When data were homoscedastic and followed a normal distribution, they were analysed by Student’s *t*-test and one-way ANOVA. Mann-Whitney and Kruskal-Wallis tests were used when data did not follow a normal distribution.

## 5. Conclusions

We describe for the first time that USP48 is a basal body-associated protein in retinal pigment epithelium cells. In the retina, USP48 is highly expressed in cones and its interaction proteome is enriched in cytoskeletal and cilium-related proteins, among them the IRD genes ARL3 and UNC119a. USP48 stabilises ARL3 and UNC119a protein levels using different mechanisms. We propose *USP48* as a potential candidate gene for unsolved inherited retinal dystrophies.

## Figures and Tables

**Table 1:** List of USP48 interactors causative of IRDs and/or ciliopathies, indicating Uniprot ID, gene name and associated human disorders ^1,2,3,4^.

Protein	Uniprot ID	Gene Name	Associated Disorders
Retinal-specific phospholipid-transporting ATPase ABCA4	O35600	*Abca4*	CRD, MD, RP, STGD
ADP-ribosylation factor-like GTPase 3	Q9WUL7	*Arl3*	RP, CRD, JBTS
Chaperonin-containing T-complex protein 1 subunit 2	P80314	*Cct2*	LCA
Crystallin Mu	O54983	*Crym*	NSD
Dynactin 2	Q99KJ8	*Dctn2*	CMT
Cytoplasmic dynein 1 heavy chain 1	Q9JHU4	*Dync1h1*	CMT
α-glucosidase II	Q8BHN3	*Ganab*	PKD
G protein subunit α-transducin 2	P50149	*Gnat2*	CRD
G protein subunit β-3	Q61011	*Gnb3*	CRD
Guanylate cyclase 2E	P52785	*Gucy2e*	CRD, LCA, RP, SLS, USH
Inosine Monophosphate dehydrogenase 1	P50096	*Impdh1*	CRD, LCA, RP
Microtubule-associated protein 1B	P14873	*Map1b*	NSD
N-myc downstream regulated 1	Q62433	*Ndrg1*	CMT with deafness
S-opsin	P51491	*Opn1sw*	Colour blindness
Phosphodiesterase 6C	Q91ZQ1	*Pde6c*	Achromatopsia, CRD, LCA
Peripherin 2	P15499	*Prph2*	CRD, LCA, MD, RP
Regulator of G protein signalling 9	O54828	*Rgs9*	Oligocone trichromacy, bradyopsia
Unc-119 lipid binding chaperone	Q9Z2R6	*Unc119a*	CRD, LCA, RP

^1^ Proteins are listed in alphabetic order. ^2^ From RetNet (https://web.sph.uth.edu/RetNet/, accessed on 22 September 2022) and Human Genome Mutation Database (HGMD, https://www.hgmd.cf.ac.uk/ac/index.php, accessed on 22 September 2022). ^3^ Inherited retinal dystrophies are written in orange, non-syndromic ciliopathies in purple and syndromic ciliopathies in green. ^4^ CMT, Charcot-Marie-Tooth disease; CORD, cone-rod dystrophy; JBTS, Joubert syndrome; LCA, Leber congenital amaurosis; MD, macular dystrophy; NSD, non-syndromic deafness; PKD, polycystic kidney disease; RP, retinitis pigmentosa; SLS, Senior-Løken syndrome; STGD, Stargardt disease; USH, Usher syndrome.

## Data Availability

The data presented in this study are available in this article and Supplementary Material.

## Supplementary Materials

The following supporting information can be downloaded at: https://www.mdpi.com/article/10.3390/ijms232012527/s1.
