# Supplementary material for: The Deubiquitinating Enzyme USP48 Interacts with the Retinal Degeneration-Associated Proteins UNC119a and ARL3
Source: Int J Mol Sci. 2022 Oct 19;23(20):12527. doi: 10.3390/ijms232012527 (PMC9603860; doi:10.3390/ijms232012527)
Supplement: Supplementary file 1 [file ijms-23-12527-s001.zip › Supplementary material/Supplementary figures.pdf]

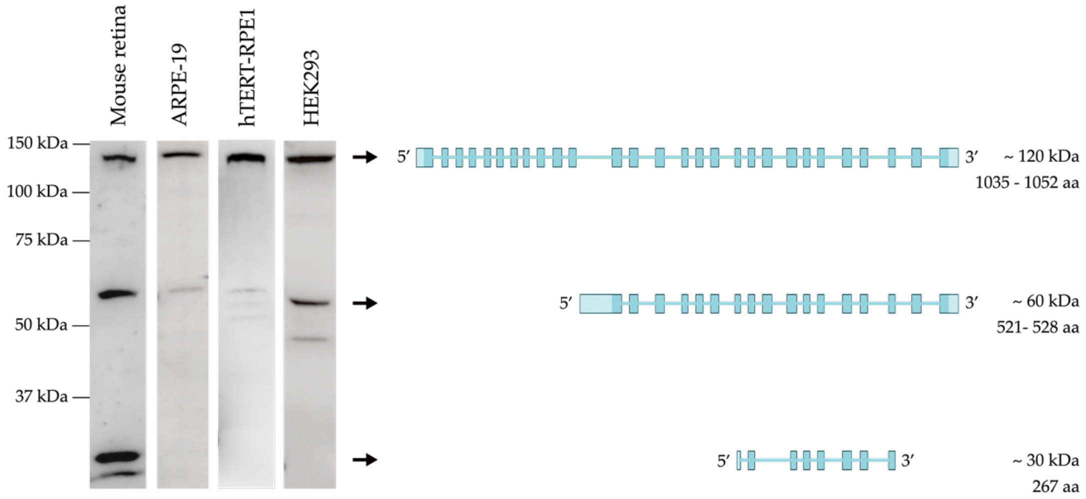


**Figure S1. Endogenous protein and mRNA USP48 isoforms detected in the mouse retina and different cultured cell lines used in this study.** Immunodetection of USP48 in protein lysates by Western blotting. Relevant protein bands that can be assigned to reported mRNA isoforms (in ENSEMBL and GenomeBrowser-UCSC) are highlighted by an arrow. The length of the protein and the calculated molecular weight are also indicated.

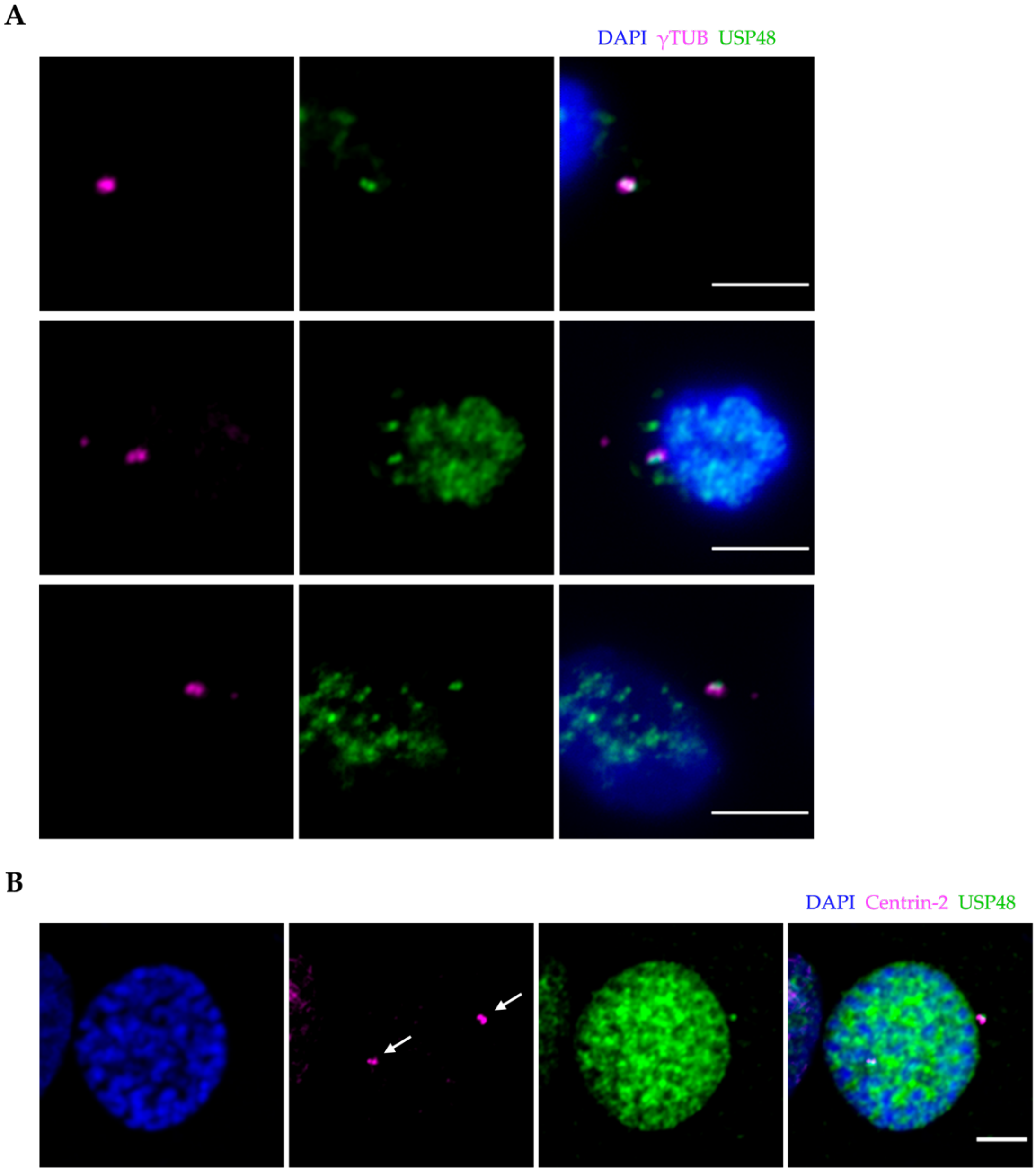


**Figure S2. Endogenous USP48 localises to the centrosome in undifferentiated human retinal pigment epithelium cells.** Undifferentiated (non-ciliated) ARPE-19 cells were immunostained with anti-γ-tubulin (γTUB, magenta) to label the centrosome and anti-USP48 (green). Cell nuclei were counterstained with DAPI (blue). Merged images reveal that a pool of the endogenously expressed USP48 co-localises with γ-tubulin in both centrioles independently of the cell cycle phase G1 in (**A**) and S-phase in (**B**), as indicated by the duplication of the centrosome (white arrows). The images are one single focal plane. Scale bar: 5 $\mu$m.

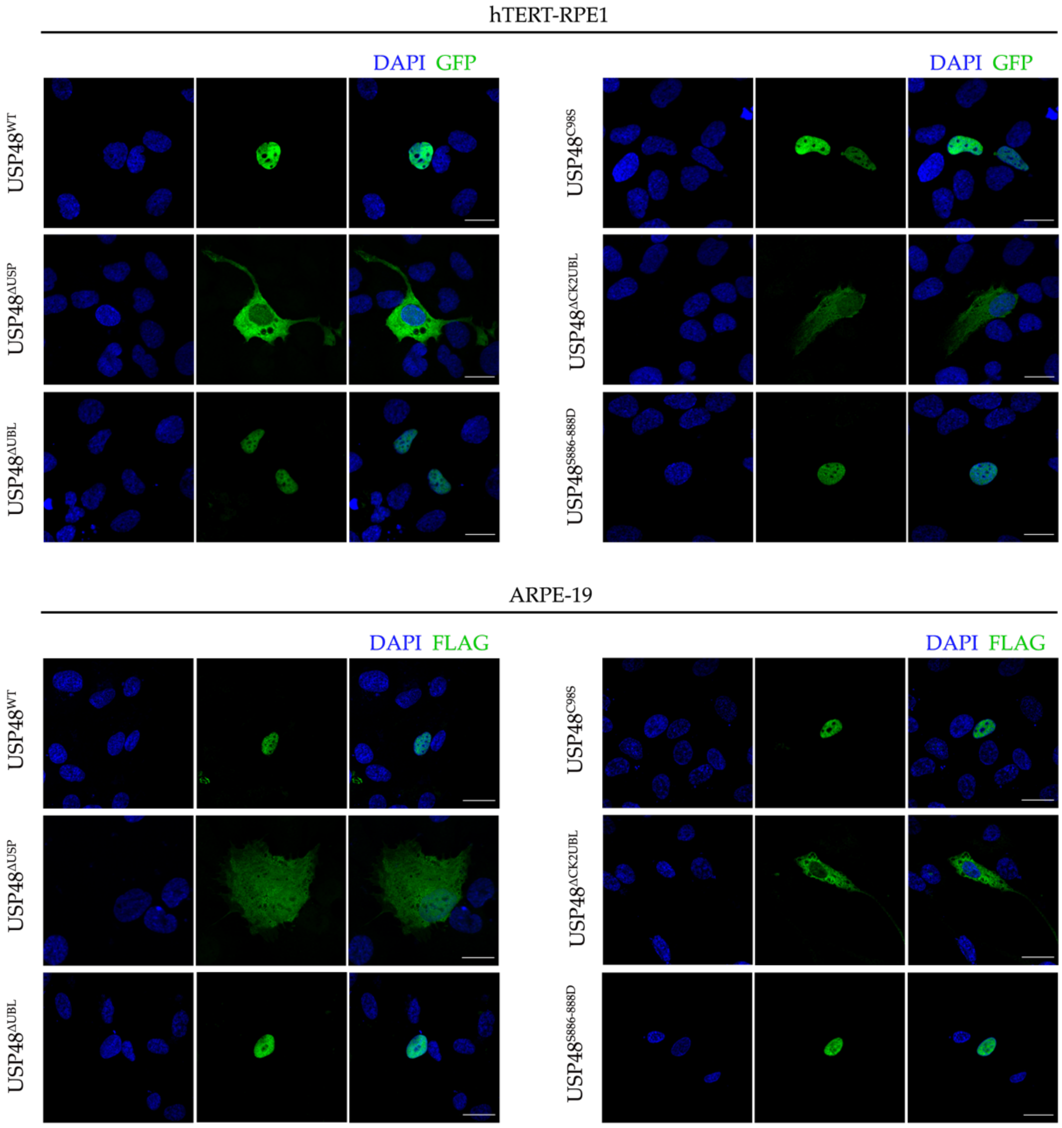


**Figure S3. Subcellular localisation of different USP48 protein mutants.** Immunofluorescence assays on transfected cells with constructs encoding USP48$^{WT}$ and the different mutant constructs show consistent subcellular localisation irrespective of the tag used (GFP or FLAG, in green) and the type of human retinal pigment epithelium cell line. Note that USP48$^{WT}$ and most mutants mainly localise at the nucleus, but the USP48$^{\Delta USP}$ and the USP48$^{\Delta CK2UBL}$ mutants show an increased localisation at the cytoplasm. Nuclei were counterstained with DAPI (blue). Scale bar: 10 $\mu$m.

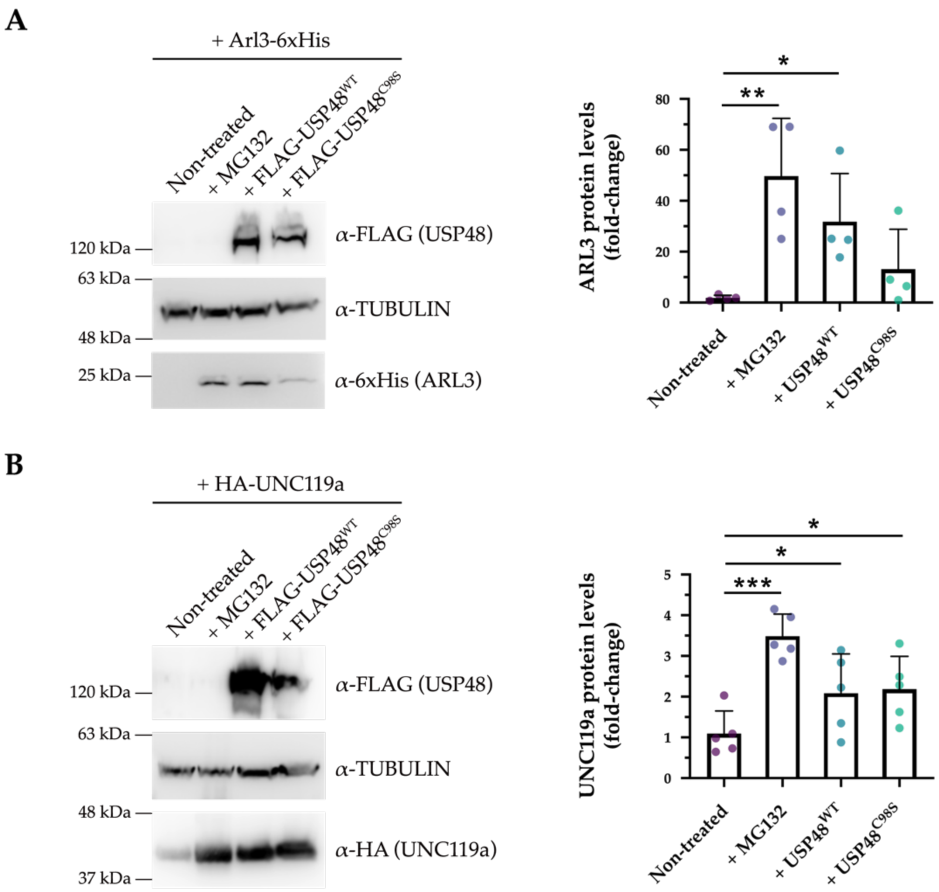


**Figure S4. USP48 stabilises ARL3 and UNC119a by different mechanisms in HEK293 cells (confirmation of assay in Figure 6 using different protein tags for ARL3 and USP48).** (**A**) ARL3-6xHis is degraded by the proteasome (rescued by MG-132 treatment) and stabilised by FLAG-USP48 in a DUB-dependent manner (one-way ANOVA; * = *p*-value ≤ 0.05, ** = *p*-value ≤ 0.01; n = 4 independent experiments). (**B**) HA-UNC119a is also degraded by the proteasome, and the rescue by FLAG-USP48 is independent of its DUB activity, since the catalytically inactive FLAG-USP48$^{C98S}$ mutant is also able to stabilise UNC119a (one-way ANOVA; * = *p*-value ≤ 0.05, *** = *p*-value ≤ 0.001; n = 5 independent replicates). All data represent the mean ± SD.
